# Systematic Review of Appropriate Robotic Intervention for Gait Function in Subacute Stroke Patients

**DOI:** 10.1155/2018/4085298

**Published:** 2018-02-06

**Authors:** Ji-Eun Cho, Jun Sang Yoo, Kyoung Eun Kim, Sung Tae Cho, Woo Seok Jang, Ki Hun Cho, Wan-Hee Lee

**Affiliations:** ^1^Department of Physical Therapy, Graduate School, Sahmyook University, Seoul, Republic of Korea; ^2^Department of Physical Therapy, Korea National University of Transportation, Chungcheongbuk-do, Republic of Korea; ^3^Department of Physical Therapy, Sahmyook University College of Health Science, Seoul, Republic of Korea

## Abstract

The purpose of this study was to critically evaluate the effects of robot-assisted gait training (RAGT) on gait-related function in patients with acute/subacute stroke. We conducted a systematic review of randomized controlled trials published between May 2012 and April 2016. This search included 334 articles (Cochrane, 51 articles; Embase, 175 articles; PubMed, 108 articles). Based on the inclusion and exclusion criteria, 7 studies were selected for this review. We performed a quality evaluation using the PEDro scale. In this review, 3 studies used an exoskeletal robot, and 4 studies used an end-effector robot as interventions. As a result, RAGT was found to be effective in improving walking ability in subacute stroke patients. Significant improvements in gait speed, functional ambulatory category, and Rivermead mobility index were found with RAGT compared with conventional physical therapy (*p* < 0.05). Therefore, aggressive weight support and gait training at an early stage using a robotic device are helpful, and robotic intervention should be applied according to the patient's functional level and onset time of stroke.

## 1. Introduction

Stroke is a common disease [1]. In most patients, disabilities remain after stroke, and long-lasting disability requires continuous management and intensive rehabilitation [1, 2]. Furthermore, the economic burden on the patient increases because of the prolonged rehabilitation period. Therefore, the application of intensive and efficient rehabilitation programs and techniques is an urgent need after stroke [3].

Gait impairment is one of the most important problems after stroke and is associated with activities of daily living and mobility issues [4]. Therefore, recovery of gait function is an important goal of rehabilitation for independent living [5]. Interventions to enhance gait function require repetitive task training with high intensity, and extensive effort by physical therapists is essential [5]. Moreover, the most effective rehabilitation intervention, including gait training, must be performed shortly after stroke and in an intensive and task-oriented manner and should include multisensory stimulation [3].

Robot-assisted gait training (RAGT) for patients in the acute/subacute stage who are nonambulatory is effective at reeducating motor control function through repetitive training of a specific task [6]; RAGT provides intensive therapy, which reduces the burden on therapists, and enhances motor reeducation with multisensory stimulation [3]. Several previous studies reported that gait training using robotic devices is effective at enhancing muscular activity patterns [7], muscle tone, joint range of motion [8], gait speed, functional gait capability [7, 9], gait independence, and mobility in the community [10, 11]. Moreover, patients who received RAGT and conventional physical therapy had a higher chance of regaining independent gait function than those who received only conventional gait training [12]. However, owing to studies that suggested RAGT is ineffective [13], the effect on gait and gait-related function in subacute stroke remains unclear. In a previous review of effectiveness in stroke patients, the RAGT group showed significant improvement in balance and balance-related activity function, but the comparison between the groups was not significant [14]. These results show that RAGT is effective, but whether it is more effective than other gait-related rehabilitation interventions is still unclear. In this context, the effect of RAGT is still not clearly demonstrated, and reviews that have recently demonstrated the effect of RAGT on gait-related outcome measures in patients with acute/subacute stroke are also limited.

Therefore, the aim of this systematic review was to investigate the effects of RAGT on acute/subacute stroke. The specific goals included identifying the effects of RAGT using assessment tools associated with gait and gait-related function in patients with acute/subacute stroke.

## 2. Methods

### 2.1. Literature Search and Study Selection

**Figure 1:**
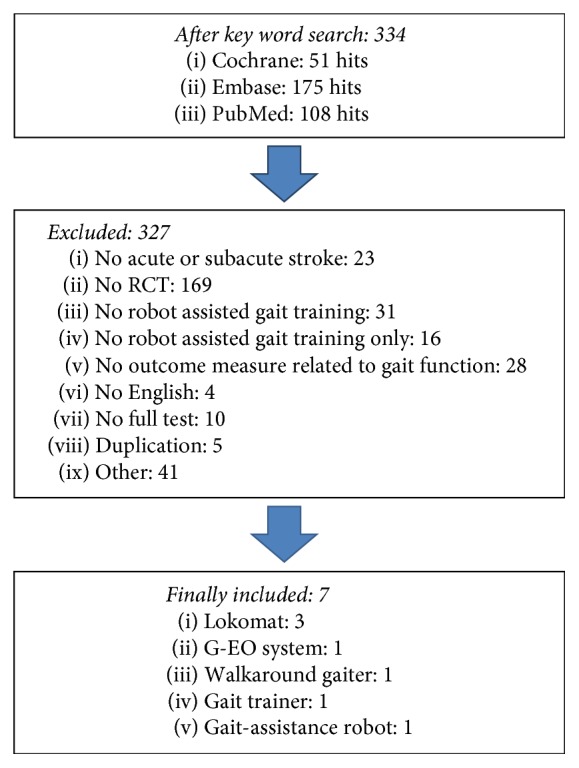
Flowchart search strategy. RCT: randomized controlled trial.

This study collected data from Cochrane, Embase, and PubMed databases from May 2012 to April 2016 to analyze the data of the last 5 years and to obtain the latest information on RAGT. We searched the data for a specific time frame to provide a recent basis for the effectiveness of RAGT for stroke patients with a specific onset of illness (subacute phase). The authors selected keywords based on the population, intervention, comparison, and outcomes (PICO) model and MeSH terms, and the search algorithm was stroke AND (robot OR robotics) AND (gait OR walking) AND rehabilitation. As a result, 51 articles from Cochrane, 175 articles from Embase, and 108 articles from PubMed were found. Four professional physical therapists analyzed the title and abstract of the articles 4 times, based on the inclusion and exclusion criteria, and 7 articles were finally selected after analyzing the full text (Figure 1). Another participant in this study confirmed the accuracy and screened for any possible omission of selected articles.

#### 2.1.1. Inclusion Criteria

1. Studies conducted on adult stroke patients aged ≥ 18 years
2. Studies conducted on patients with acute/subacute stroke (within 3 months after onset)
3. Studies that included RAGT in combination with physiotherapy (or usual care) versus physiotherapy (or usual care) as the intervention method for regaining and improving walking ability after stroke
4. Studies that used measurement tools associated with gait and gait-related function
5. Randomized clinical trial
6. Control group which received conventional rehabilitation therapy

#### 2.1.2. Exclusion Criteria

1. Study using an upper-limb robot as an intervention method
2. Studies that compared different types of robots
3. Studies on other interventions combined with RAGT except for usual care for regaining and improving walking ability after stroke
4. Studies written in languages other than English
5. Studies for which the full text was not found

### 2.2. Methodological Quality Assessment

The selected papers were analyzed with regard to methodological quality using the PEDro scale. The PEDro scale contains the following 11 items: eligibility, random allocation, concealed allocation, baseline comparability, blinded subjects, blinded therapists, blinded raters, key outcomes, intention-to-treat analysis, between-group comparison, and precision and variability. The official score of the papers described in the electronic database was used. Five professional physical therapists and researchers with at least 5 years of clinical experience evaluated each item for quality. After scoring according to each item, the authors were cross-checked and measured the score in the controversial items after discussion.

### 2.3. Data Collection

The authors systematically reviewed general characteristics, such as the number of subjects, sex, age, diagnosis, side of hemiplegia, time after stroke, intervention method, measurement tools for gait and gait-related function, and characteristics of the studies associated with the results.

### 2.4. Effect Size Calculations

Cohen's* d* was applied for effect size calculation by using the difference between 2 means divided by the pooled standard deviations. First, pretest, posttest, and follow-up between-group comparisons were analyzed. Second, intragroup comparisons were analyzed for pretest versus posttest, posttest versus follow-up, and pretest versus follow-up results. Effect sizes ranging from 0.2 to 0.5, from 0.5 to 0.8, and from 0.8 to infinity were defined as small, medium, and large, respectively [20].

## 3. Results

### 3.1. Study Quality Evaluation

Quality evaluation was performed based on the PEDro score suggested for evidence-based review of stroke rehabilitation. The final score was settled when 3 of the 4 authors reached agreement after repeated review and analysis. All 7 studies conducted randomized trials, and the PEDro score ranged from 7 to a maximum of 10, with a mean of 8.28 (Table 1).

### 3.2. General Characteristics of the Study Population

The authors analyzed the therapeutic effects of RAGT by reviewing the 7 articles. Among 220 subjects, 112 received RAGT and 108 were included in the control group. The male participants comprised 61.05% (female, 38.95%), and the patients with left and right paralysis comprised 53.85% and 46.15%, respectively, of the study subjects. The mean progression time after the onset of stroke was between 16.1 and 116.2 days, all of which were included in the acute/subacute stage, and the age range of the subjects was 40.4 to 80 years (Table 2).

### 3.3. Descriptive Analysis

Table 2 presents the characteristics of the included studies. The gait and gait-related function outcomes used in the selected studies were functional ambulation classification (FAC), gait velocity, timed up-and-go (TUG) time, 6-minute walk test (6 MWT), 10-minute walking test (TWT), Tinetti gait scale, Rivermead nobility index (RMI), Berg balance scale (BBS), Barthel index (BI), and functional independence measure (FIM). The mean test results with standard deviations and the effect size calculations are reported in Table 3. Only the effect sizes in 5 studies [3, 4, 16, 17, 19] were calculated after excluding those that showed only posttraining differences without pretest and posttest means and standard deviations [15] and those that included medians and ranges only [18].

### 3.4. Outcome Measures: Gait and Gait-Related Function

The most commonly used assessment tools for gait and gait-related function included the TWT, which is used to assess walking speed or gait velocity; FAC, which was used in 5 studies; RMI, which was used in 3 studies; and FIM and BI, which were used as assessment tools in 2 of the 7 studies. The BBS, 6 MWT, and TUG test were used as assessment tools in 1 of the studies associated with gait (Table 3).

### 3.5. Intervention Effects: Within- and between-Group Differences

The period of RAGT ranged from 2 to 5 weeks, with varied intervention durations ranging from 400 to 960 minutes. Five studies [4, 15–17, 19] (71.43%) reported intervention effects after the follow-up period. Conventional rehabilitation therapy conducted in control groups included general gait training, muscle strength exercise, Bobath approach therapy, gait training on parallel bars, and stair-ascent activity.

All experimental groups in the studies received interventions associated with RAGT. Three (42.85%) of the studies used Lokomat as an intervention method [3, 17, 19], and 4 other studies used the G-EO system [15], walk-around gaiter [4], gait trainer (GT) [16], and gait-assistance robot (GAR) [18] (14.28% each).

In a study that used Lokomat as an intervention method, Chang et al. [3] reported that the experimental group, which received RAGT, showed no significant change in FAC (small effect size). Furthermore, Van Nunen et al. [17] reported that both the experimental group (*p* < 0.01, medium effect size) and the control group (*p* < 0.01, small effect size) showed significant improvements in gait speed after 10 weeks of intervention and at 24 and 36 weeks during the follow-up period as compared with baseline values but found no significant difference between the groups (posttest: small effect size, follow-up: median effect size). In addition, both groups showed significant improvements in FAC (*p* < 0.01, large effect size), BBS score (*p* < 0.01, small effect size), RMI (*p* < 0.01, large effect size), and TUG time, but no significant difference was found between the groups (posttest: small effect size). Taveggia et al. [19], in another study on the effects of Lokomat, reported significant improvements in gait speed (*p* < 0.05, large effect size) and FIM (*p* < 0.05, medium effect size) after 5 weeks of intervention and at follow-up after 17 weeks, but no significant difference was found between the groups (posttest: median effect size). In other studies that used other robots as intervention methods, Morone et al. [16] assessed motor function in stroke patients by using the Motricity index and divided the patients into 2 groups, that is, one consisting of those with good motor function and the other consisting of those with poor motor function. They conducted 4 weeks of intervention by using the gait trainer with follow-up for 3 months and found significant differences in FAC (*p* = 0.001, large effect size), BI (*p* = 0.005, large effect size), and RMI (*p* = 0.001, large effect size) between the RAGT group with poor motor function and the control group alone. This difference remained significant after the follow-up period. During the follow-up period, significant differences were found in FAC (*p* = 0.002, large effect size), BI (*p* = 0.024, large effect size), and RMI (*p* = 0.010, large effect size) between the RAGT group with poor motor function and the control group. Hesse et al. [15] conducted gait and stair-ascent training by using the G-EO system and found that both the RAGT group and control group showed significant improvements in FAC, gait speed, and RMI (*p* < 0.001). In addition, the RAGT group showed substantial improvements in FAC, gait speed, and RMI after the intervention in the between-group comparison; the improvement in FAC continued after follow-up. Dragin et al. [4] conducted studies using a walk-around gaiter as an intervention for 4 weeks with follow-up for 6 months and reported a significant difference in BBS score in both groups after follow-up (*p* < 0.05, small effect size) and that the gait speed in the RAGT group remained significant after the intervention and follow-up (*p* < 0.05, large effect size). In the between-group comparison, gait speed (large effect size) and BBS score (small effect size) remained significant after 4 weeks and only gait speed remained significant after 6 months of follow-up (large effect size). In a study by Ochi et al. [18], in which subjects received treadmill gait training using GAR, both groups showed significant improvements in FAC (*p* < 0.01) and FIM (*p* < 0.01) mobility score, and the RAGT group showed significant improvement in FAC in the between-group comparison (*p* = 0.02).

## 4. Discussion

The purpose of this study was to investigate the effects of RAGT on gait and gait-related function and to investigate up-to-date evidence for an effective robotic intervention method for patients with acute/subacute stroke.

Recent evidence suggests that intensive stroke rehabilitation is effective when performed in the early stage and should be task-specific, with multisensory stimulation [21, 22]. This is associated with brain plasticity; the best time for boosting plasticity-dependent recovery is within 3 months after the stroke event [23]. Robotic rehabilitation provides intensive, task-oriented, repeated work with the supervision or help of a therapist and can be utilized as a tool for stroke rehabilitation [24].

A common advantage of RAGT is that it partially or totally supports body weight bearing and allows high-intensity, complex gait cycle training for nonambulatory patients, which is difficult for the therapist to achieve alone. Body weight support through a robotic device facilitates gait recovery in nonambulatory patients [25]. In addition, RAGT relieves the therapist's burden, ensures patient safety by preventing falling during training, and provides constant and repeatable training. These advantages have important implications in terms of the physiotherapist's work efficiency with regard to application of interventions and the quality of care provided [26]. A recent review article suggested that electromechanical- and robotic-assisted gait training is more effective for nonambulatory stroke patients than for ambulatory stroke patients [27]. In this study, Morone et al. reported the effect of a gait trainer in nonambulatory patients with subacute stroke according to the level of impairment [16]. Patients with greater motor impairment showed significant changes in independent walking ability, independence for activities of daily living, and balance and exercise abilities at discharge and at 2-year follow-up [16]. In other studies by Morone and colleagues, the robotic device was more effective than conventional therapy in patients with more severe impairments and provided higher intensity of treatment [16, 28]. Moreover, because the robotic device helped to restore gait by providing external support, benefit was maintained until recovery of the ability to walk over ground unsupported [22]. These results provide a basis for determination of who will gain more from RAGT. The evidence suggests that RAGT is effective for patients with subacute, nonambulatory, and higher functional impairment after stroke. Another advantage of body weight support through RAGT is that it allows patients with severe neurological impairment to experience early verticality, thereby reducing energy consumption and cardiorespiratory load [29]. This is related to the quality of life in stroke patients with cardiovascular diseases [30]. Thus, RAGT provides not only simple and repetitive movement but also generates more complex, controlled multisensory stimulation [31]. Another feature of RAGT that cannot be replaced by conventional gait training is the quantitative evaluation of several parameters related to patient performance (e.g., range of motion, walking speed, spasticity, and muscle strength) through the robotic device.

Robotic use for walking rehabilitation can be classified according to the method applied to the body. For instance, “exoskeletal robots” move hip, knee, and ankle joints and apply control during the gait cycle, whereas “end-effector robots” move only the feet and are often placed on a support (footplate) that simulates the stance and swing phases [32]. Of the 5 robots discussed in this review, the Lokomat [3, 17, 19] is a typical exoskeletal robot, whereas the G-EO system [15], walk-around gaiter [4], gait trainer [16], and GAR [18] are end-effector robots.

Lokomat, which is generally used in gait rehabilitation, is a robot-assisted gait device combined with a harness-supported body weight system, used in combination with a treadmill. The legs of the robot are controlled by a computer, whereas the hip and knee joints are fixed on an exoskeletal device for training [13, 33]. The patient's hip (hip, knee, and ankle joints) is fixed to the device and moves rhythmically according to the preprogrammed gait kinematic pattern [8]. However, a number of previous studies suggested that gait training that maximizes the level of support in the motor pattern of gait, such as Lokomat, does not always produce a positive result and that an appropriate intervention might be more effective [13, 34]. In one study included in this review, walking speed, FAC, BBS score, and RMI were significantly improved in the group with Lokomat training, but the control group also showed significant improvements and the result of the between-group comparison was not significant (small effect size) [17]. Another study using Lokomat RAGT showed a significant effect on gait function in posttraining and follow-up (large effect size), but the comparison between groups was not significant [19]. Another study did not report any significant effects on gait function after RAGT [3]. In recent years, the gait trainer (GT II; Rehastim, Berlin, Germany) and end-effector-type RAGT devices allow patients to place their feet on the footplate and modulate movement of the feet during the stance and swing phases [35]. The feet are always in contact with the platform for modulation of the gait pattern [36]. The striking feature of the GAR is that it minimizes body weight support, allowing the patient to experience spontaneous trunk control and weight bearing during gait. [37]. The G-EO system is comprised of a footplate designed to enable gait and stair-ascent activity [38]. Thus, this device is effective in reducing the risk of falls, which can occur during stair gait training, and the therapist's burden [39]. In this review, we also reported a significant improvement in gait function with the end-effector-type robot in the between-group comparison (median to large effect size). End-effector robots allow patients to extend their knees with more freedom. In addition, the task of maintaining balance, which allows more body weight bearing, may be more demanding in using end-effector robots. This advantage influenced improvement of gait-related function in stroke patients. However, in this review, functional levels varied (FAC ranged from 0 to 2) but all patients were at a nonfunctional or dependent level, and the duration and intensity of the intervention were also different. This was an important factor that affected the results of the study; therefore, the effect of the robot is difficult to determine on the basis of the results of this review.

A Cochrane review in 2013 analyzed 23 randomized trials that were conducted among 999 stroke patients and reported that patients who receive electromechanical-assisted gait training in combination with physiotherapy after stroke were more likely to achieve independent walking than those who received gait training without these devices [40]. In addition, the authors reported that patients in the acute phase as well as those who are nonambulatory may benefit from this type of training. Moreover, in another study, significant effects on gait speed, gait ability, and muscle power were observed in patients with subacute stroke with gait impairment after RAGT, as compared with on-ground gait training [37]. However, another recent systematic review reported that RAGT improved balance function in patients with subacute and chronic stroke, but the improvement was not statistically significant [14]. A recent Cochrane review including 36 studies and 1,472 stroke patients found that RAGT was most effective for patients with stroke who could not walk, as well as during the first 3 months after stroke. As a result of this review, gait training using electromechanical robots in subacute stroke patients proved to be significant [4, 15–19]. While some studies showed a significant difference between a control group and an experimental group that performed general gait training [4, 15, 16, 18], other studies did not [3, 17, 19]. Therefore, it cannot be concluded that RAGT is more effective than general gait training. However, the benefits of RAGT and its merits are obvious, enabling physical therapists to maximize their effectiveness in improving gait ability in subacute stroke patients when RAGT is combined with conventional therapy.

This review has several limitations. It included a small number of studies (and subjects). Future review studies should include a qualitative analysis of the frequency and intensity of interventions, including more studies on subacute stroke patients. Further studies are needed to demonstrate the effectiveness of RAGT according to the functional level of stroke patients in not only the subacute phase but also the chronic phase.

## Figures and Tables

**Table 1:** Scores of methodological quality assessment of the included studies.

PEDro	Hesse et al., 2012 [15]	Morone et al., 2012 [16]	Chang et al., 2012 [3]	Dragin et al., 2014 [4]	Van Nunen et al., 2015 [17]	Ochi et al., 2015 [18]	Taveggia et al., 2016 [19]
	Randomized controlled trial	Randomized controlled trial	Randomized controlled trial	Randomized controlled trial	Randomized controlled trial	Randomized controlled trial	Randomized controlled trial
Eligibility	Y	N	Y	Y	Y	Y	Y
Randomized allocation	Y	Y	Y	Y	Y	Y	Y
Concealed allocation	N	Y	Y	N	N	Y	Y
Baseline comparability	Y	Y	Y	Y	Y	Y	Y
Blinded subject	N	N	N	N	N	Y	N
Blinded therapists	N	N	N	N	N	N	Y
Blinded raters	Y	N	Y	N	N	Y	Y
Key outcomes	Y	Y	Y	Y	Y	Y	Y
Intention to treat	Y	Y	Y	Y	Y	Y	Y
Comparison between groups	Y	Y	Y	Y	Y	Y	Y
Precision and variability	Y	Y	Y	Y	Y	Y	Y
	8/11	7/11	9/11	7/11	7/11	10/11	10/11

Y: yes. N: no.

**Table 2:** Descriptive analysis of the included studies.

	Population		Intervention		Comparison	Outcome
	Participants in experimental group	Participants in control group	Training frequency	Intervention group	Control group	Gait & gait-related activities results
Hesse et al., 2012 [15]	*n* = 15, 9 F/6 M Age: 63.7 ± 9.4 Diagnosis (hemorrhage/ischemic): 4/11 Side of hemiplegia: 6 L/14 R *Weeks after stroke: 5.7 ± 2.3*	*n* = 15, 9 F/6 M Age: 66.4 ± 11.9 Diagnosis (hemorrhage/ischemic): 5/110 Side of hemiplegia: 6 L/14 R *Weeks after stroke: (week): 5.1 ± 1.6*	60 min/session, for 4 week (total 20 sessions and 10 hours RAGT)	30 min of robot-assisted gait and stair climbing training *(G-EO system)* + 30 min physiotherapy	60 min of conventional physiotherapy gait and stair climbing therapy	(1) Within groups: significant in FAC, gait velocity, RMI in both groups during intervention and follow-up (2) Between groups: significant in FAC between groups after intervention

Morone et al., 2012 [16]	(HM) *n* = 12 Age: 68.33 ± 9.11 Diagnosis (hemorrhage/ischemic): 9/3 Side of hemiplegia: 8 L/4 R *Days after stroke: Mean of 20 days* (LM) *n* = 12 Age: 55.58 ± 13.35 Diagnosis (hemorrhage/ischemic): 9/3 Side of hemiplegia: 3 L/9 R *Days after stroke: Mean of 20 days*	(HM) *n* = 12 Age: 62.92 ± 17.43 Diagnosis (hemorrhage/ischemic): 12/0 Side of hemiplegia: 4 L/8 R *Days after stroke:* *Mean of 20 days* (LM) *n* = 12 Age: 60.17 ± 9.59 Diagnosis (hemorrhage/ischemic): 11/1 Side of hemiplegia: 5 L/7 R *Days after stroke: Mean of 20 days*	2 sessions/day, 5 days/week, for 4 weeks (total 20 sessions and 10 hours RAGT)	Robotic-assisted gait training *(gait trainer)* + conventional therapy	Conventional gait training	(1) Between groups: significant in FAC, BI, RMI between 2 LM groups (2) Between groups: no significance between the 2 HM groups

Chang et al., 2012 [3]	*n* = 20, 13 M/7 F Age: 55.5 ± 12.0 Diagnosis (hemorrhage/ischemic): 8/12 side of hemiplegia: 6 R/14 L *Days after stroke: 16.1 ± 4.9*	*n* = 17, 10 M/7 F Age: 59.7 ± 12.1 Diagnosis (hemorrhage/ischemic): 6/11 side of hemiplegia: 6 R/11 L *Days after stroke: 18.2 ± 5.0*	2 sessions/day, 5 day/week for 2 weeks (total 20 sessions and 13.3 hours RAGT)	40 min gait training *(Lokomat)* + 60 min conventional physical therapy	100 min conventional physical therapy	(1) Between groups: both groups not significant in FAC

Dragin et al., 2014 [4]	*n* = 11, 9 M/2 F Age: 57.3 ± 10.9 Diagnosis (hemorrhage/ischemic): 2/9 Side of hemiplegia: 5 L/6 R *Days after stroke: 38 ± 21*	*n* = 11, 9 M/2 F Age: 58.1 ± 11.4 Diagnosis (hemorrhage/ischemic): 1/10 Side of hemiplegia: 7 L/4 R *Days after stroke: 36 ± 21*	30 min/session, 5 sessions/week, for 4 weeks (total 20 sessions and 10 hours RAGT)	Walked assisted by *Walkaround* at a pace in a range from 0.3 to 0.7 m/s	Walk at their natural pace and use conventional gait assistance (cane, physical therapist assistance)	(1) Within groups: significant for the BBS after 6 months in both groups and in gait speed among the Exp group at the end of therapy and after 6 months (2) Between groups: significant for the gait speed and BBS after 4 weeks and in gait speed after 6 months between groups

Van Nunen et al., 2015 [17]	*n* = 16, 10 M/6 F Age: 50.0 ± 9.6 Diagnosis (hemorrhage/ischemic): 9/7 Side of stroke: 5 R/11 L *Days after stroke: 61.6 ± 28.7*	*n* = 14, 5 M/8 F Age: 56.0 ± 8.7 Diagnosis (hemorrhage/ischemic): 10/4 Side of stroke: 5 R/9 L *Days after stroke: 67.1 ± 49.1*	3.5 h/week, for 8 weeks (total 16 hours RAGT)	*Lokomat* (2 h) + conventional therapy (1.5 h) a week aimed at improving walking ability	Conventional therapy (3.5 h) of physical therapy a week aimed at improving walking ability	(1) Within groups: significant for walking speed, FAC, BBS, RMI, TUG after training and follow-up in both groups (2) Between groups: no significant differences in improvements in any of the variables between groups

Ochi et al., 2015 [18]	*n* = 13, 11 M/2 F Age: 61.8 ± 7.5 Diagnosis (hemorrhage/ischemic): 8/5 Side of hemiplegia: 7 R/6 L *Days after stroke: 22.9 ± 7.4*	*n* = 13, 9 M/4 F Age: 65.5 ± 12.1 Diagnosis (hemorrhage/ischemic): 8/5 Side of hemiplegia: 8 R/5 L *Days after stroke: 26.1 ± 8.0*	80 min/session, 5 sessions/week for 4 weeks (total 20 sessions and 6.7 hours RAGT)	20 min of *GAR-assisted gait training* + 60 min of standard physical therapy	20 min of overground conventional gait training + 60 min of standard physical therapy	(1) Within groups: significant for FAC and FIM after training in both groups (2) Between groups: significant group difference in improvement in FAC after training

Taveggia et al., 2016 [19]	*n* = 13, 7 M/6 F Age: 71 ± 5 *Days after stroke: 60.1 ± 49.5*	*n* = 15, 10 M/5 F Age: 73 ± 7 *Days after stroke: 39.4 ± 31.7*	90 min/day, 5 days/week, for 5 weeks (total 12.5 hours RAGT)	Bobath approach (60 min) + robotic gait training on the *Lokomat* robotic system (30 min)	Bobath approach (60 min) + activities targeted at improvement in walking (30 min)	(1) Within group: significant increase in TWT at the end of the treatment and follow-up in experimental group (2) Between groups: no significant differences in improvements in any of the variables between groups

Con: control group. Exp: experimental group. FAC: functional ambulation classification. RMI: Rivermead mobility index. HM: high Motricity. LM: low Motricity. BI: Barthel index. BBS: Berg balance scale. TWT: 10 m walking test. TUG: timed up and go. 6 MWT: 6 m walk test. FIM: functional independence measure. RAGT: robot assisted gait training.

**Table 3:** Mean value and effect size calculations on gait and gait related function outcomes.

Outcomes	Groups						Difference within groups				Cohen's *d*								
	Pretraining		Posttraining		Follow-up		Post minus pre		Follow-up minus pre		Exp-Con			Pre-post		After follow-up		Before follow-up	
	Exp	Con	Exp	Con	Exp	Con	Exp	Con	Exp	Con	Pre	Post	Follow-up	Exp	Con	Exp	Con	Exp	Con
*Hesse et al., 2012 [15]*																			
FAC	1.5 (0.5)	1.4 (0.5)	N/A	N/A	N/A	N/A	2.4 (1.2)^*∗*†^	1.2 (1.5)^*∗*^	3.0 (0.8)^*∗*^	1.7 (1.8)^*∗*^	0.2	N/A	N/A	N/A	N/A	N/A	N/A	N/A	N/A
Gait velocity	0.27 (0.12)	0.25 (0.08)	N/A	N/A	N/A	N/A	0.31 (0.17)^*∗*^	0.16 (0.20)^*∗*^	0.39 (0.22)^*∗*^	0.25 (0.32)^*∗*^	0.19	N/A	N/A	N/A	N/A	N/A	N/A	N/A	N/A
RMI	3.9 (1.1)	3.7 (1.1)	N/A	N/A	N/A	N/A	3.8 (2.2)^*∗*^	2.1 (1.8)^*∗*^	5.8 (3.2)^*∗*^	3.7 (3.4)^*∗*^	0.18	N/A	N/A	N/A	N/A	N/A	N/A	N/A	N/A
*Morone et al., 2012 [16]*																			
FAC (LM)	0.1 (0.3)	0.0 (0.0)	4.0 (0.9)^†^	2.1 (1.2)	4.7 (0.5)^†^	3.1 (1.3)	N/A	N/A	N/A	N/A	N/A	1.79^¶^	1.62^¶^	5.81^¶^	N/A	0.96^¶^	0.80	11.16^¶^	N/A
RMI (LM)	1.6 (0.8)	1.3 (0.9)	9.4 (2.7)^†^	4.9 (2.0)	11.8 (3.5)^†^	7.0 (3.6)	N/A	N/A	N/A	N/A	0.35	1.89^¶^	1.35^¶^	3.92^¶^	2.32^¶^	0.77	0.72	4.02^¶^	2.17^¶^
BI (LM)	14.2 (11.8)	7.9 (8.9)	69.6 (15.1)^†^	52.1 (14.1)	76.9 (11.5)^†^	64.7 (14.0)	N/A	N/A	N/A	N/A	0.60	1.19^¶^	0.95^¶^	4.08^¶^	3.74^¶^	0.54	0.90^¶^	5.38^¶^	4.84^¶^
FAC (HM)	0.0 (0.0)	0.0 (0.0)	3.8 (1.1)	3.8 (1.1)	4.3 (0.9)	4.3 (0.9)	N/A	N/A	N/A	N/A	N/A	0	0	N/A	N/A	0.50	0.50	N/A	N/A
RMI (HM)	1.8 (1.4)	2.2 (1.9)	7.4 (4.1)	10.1 (4.0)	10.4 (3.6)	10.6 (3.9)	N/A	N/A	N/A	N/A	0.23	0.66	0.05	1.83^¶^	2.52^¶^	0.78	0.13	3.15^¶^	2.74^¶^
BI (HM)	20.0 (17.2)	24.6 (15.3)	64.2 (21.2)	74.2 (20.3)	74.3 (18.7)	77.6 (20.4)	N/A	N/A	N/A	N/A	0.28	0.48	0.16	2.29^¶^	2.76^¶^	0.51	0.17	3.02^¶^	2.94^¶^
*Chang et al., 2012 [3]*																			
FAC	0.5 (0.5)	0.4 (0.5)	1.3 (0.7)	1.4 (0.8)	N/A	N/A	N/A	N/A	N/A	N/A	0.2	0.13	N/A	0.32	1.50^¶^	N/A	N/A	N/A	N/A
*Dragin et al., 2014 [4]*																			
Gait speed	0.38 (0.09)	0.37 (0.10)	0.54 (0.13)^*∗*†^	0.44 (0.09)	0.59 (0.11)^*∗*†^	0.45 (0.10)	0.16 (0.1)^*∗*^	0.07 (0.1)	0.21 (0.1)^*∗*^	0.08 (0.1)	0.10	0.89^¶^	1.33^¶^	1.43^¶^	0.74	0.42	0.11	2.09^¶^	0.80
BBS	38.4 (9.2)	36.4 (12.0)	44.8 (7.9)^†^	40.5 (10.1)	46.7 (8.1)^*∗*^	43.9 (9.7)^*∗*^	6.4 (3.1)	4.18 (3.34)	8.4 (2.2)^*∗*^	7.55 (4.76)^*∗*^	0.18	0.47	0.31	0.75	0.37	0.24	0.34	0.96^¶^	0.69
BI	78.6 (10.9)	75.4 (14.6)	87.3 (7.5)	85.9 (8.6)	90.9 (6.6)	91.4 (6.4)	8.6 (6.4)	10.45 (7.57)	12.3 (6.8)	15.91 (12.81)	0.24	0.17	0.07	0.93^¶^	0.88^¶^	0.51	0.73	1.37^¶^	1.42^¶^
*Van Nunen et al., 2015 [17]*																			
Walking speed	0.20 (0.16)	0.17 (0.17)	0.31 (0.27)^*∗*^	0.21 (0.21)^*∗*^	0.39 (0.30)^*∗*^	0.26 (0.21)^*∗*^	N/A	N/A	N/A	N/A	0.18	0.41	0.50	0.50	0.21	0.28	0.24	0.79	0.47
FAC	1.25 (0.58)	0.29 (0.99)	2.00 (0.88)^*∗*^	2.00 (0.13)^*∗*^	2.60 (0.84)^*∗*^	2.27 (1.42)^*∗*^	N/A	N/A	N/A	N/A	1.18^¶^	0	0.28	1.01^¶^	2.42^¶^	0.70	0.27	1.87^¶^	1.62^¶^
BBS	14.4 (9.5)	15.0 (9.6)	17.4 (14.7)^*∗*^	17.9 (10.2)^*∗*^	21.7 (12.2)^*∗*^	20.4 (12.0)^*∗*^	N/A	N/A	N/A	N/A	0.06	0.03	0.10	0.24	0.29	0.32	0.22	0.67	0.50
RMI	3.8 (2.0)	3.8 (2.0)	5.8 (2.3)^*∗*^	5.6 (2.4)^*∗*^	6.5 (2.2)^*∗*^	6.1 (2.5)^*∗*^	N/A	N/A	N/A	N/A	0	0.08	0.16	0.93^¶^	0.81^¶^	0.31	0.20	1.28^¶^	1.02^¶^
TUG	45	41	47	42	32	37	N/A	N/A	N/A	N/A	N/A	N/A	N/A	N/A	N/A	N/A	N/A	N/A	N/A
*Ochi et al., 2015 [18]*																			
FAC	1 (1-2)	2 (1-2)	3 (3-4)^*∗*†^	3 (3-3)^*∗*^	N/A	N/A	N/A	N/A	N/A	N/A	N/A	N/A	N/A	N/A	N/A	N/A	N/A	N/A	N/A
Walking speed	N/A	N/A	0.26 (0.18–0.70)	0.17 (0.15–0.24)	N/A	N/A	N/A	N/A	N/A	N/A	N/A	N/A	N/A	N/A	N/A	N/A	N/A	N/A	N/A
FIM	7 (6–10)	7 (7–9)	13 (13–21)^*∗*^	13 (12–17)^*∗*^	N/A	N/A	N/A	N/A	N/A	N/A	N/A	N/A	N/A	N/A	N/A	N/A	N/A	N/A	N/A
*Taveggia et al., 2016 [19]*																			
6 MWT	124.8 (117.6)	171.4 (130.0)	191.6 (174.8)	272.8 (155.6)	184.9 (139.8)	295.6 (183.9)^*∗*^	66.8 (30.3)	101.4 (40.6)	59.6 (27.5)	124.2 (36.9)^*∗*^	0.37	0.49	0.67	0.45	0.71	0.04	0.13	0.47	0.78
TWT	0.27 (0.25)	0.46 (0.26)	0.56 (0.44)^*∗*^	0.66 (0.19)	0.53 (0.37)^*∗*^	0.72 (0.38)	0.28 (0.08)^*∗*^	0.21 (0.1)	0.25 (0.07)^*∗*^	0.26 (0.09)	0.74	0.29	0.50	0.81^¶^	0.88^¶^	0.07	0.20	0.82^¶^	0.80
Tinetti gait	3.3 (2.9)	5.2 (1.9)	5.4 (2.7)	8.6 (3.8)	5.8 (2.9)	8.6 (1.9)	2.1 (0.6)^*∗*^	3.4 (0.8)^*∗*^	2.4 (0.4)^*∗*^	3.4 (0.6)^*∗*^	0.77	0.97^¶^	1.14^¶^	0.75	1.13^¶^	0.14	0.00	0.86^¶^	1.79^¶^
FIM	75.6 (22.8)	90.8 (15.3)	89.4 (24.3)	100.2 (11.0)	100.1 (21.8)	100.6 (9.9)	13.8 (3.3)^*∗*^	9.4 (4.6)	24.5 (4.4)^*∗*^	12.8 (6.2)	0.78	0.57	0.02	0.59	0.71	0.46	0.04	1.10^¶^	0.76

Exp: experimental group. Con: control group. FAC: functional ambulation classification. RMI: Rivermead mobility index. HM: high Motricity. LM: low Motricity. BI: Barthel index. BBS: Berg balance scale. TWT: 10 m walking test. TUG: timed up and go. 6 MWT: 6 m walk test. FIM: functional independence measure. N/A: not available. ^*∗*^Significantly different within groups, *p* < 0.05; ^†^significantly different between groups, *p* < 0.05; ^¶^large effect size.
